# Impact of Sourdough from a Commercial Starter Culture on Quality Characteristics and Shelf Life of Gluten-Free Rice Breads Supplemented with Chickpea Flour

**DOI:** 10.3390/foods13142300

**Published:** 2024-07-22

**Authors:** Sevasti Keramari, Chrysanthi Nouska, Magdalini Hatzikamari, Costas G. Biliaderis, Athina Lazaridou

**Affiliations:** Department of Food Science and Technology, School of Agriculture, Aristotle University of Thessaloniki, P.O. Box 235, 54124 Thessaloniki, Greece; sevikeramari@gmail.com (S.K.); cinouska@agro.auth.gr (C.N.); magdah@agro.auth.gr (M.H.); biliader@agro.auth.gr (C.G.B.)

**Keywords:** batter back extrusion test, batter acidity, bread acidity, crumb texture, bread staling, bread sensory evaluation

## Abstract

This study aimed to develop a novel gluten-free bread using a rice/chickpea flour-based sourdough, fermented by a commercial starter culture, to improve the quality characteristics and shelf life of this product. The effects of sourdough incorporation, chickpea flour content (6.5 and 10.0%), and added water level (80–110%) on batter rheology and bread quality were investigated; bread textural characteristics upon storage (0–2 days) were also monitored. The level of added water was the primary factor influencing batter rheology, as evaluated by the back extrusion test. Sourdough incorporation decreased the pH and increased the acidity of batters and breads. The inclusion of sourdough, the water level, and the storage time affected the moisture and texture parameters of the bread crumb. Sourdough incorporation into bread formulations decreased crumb hardness and staling rate and increased loaf specific volume. Moreover, intermediate water (90 and 100%) and high chickpea (10%) levels in the batters increased loaf specific volumes and crust redness, respectively. Sensory analysis revealed that sourdough-enriched breads were preferred by the assessors concerning general appearance and crumb texture. Overall, bread formulations with the incorporation of sourdough, at a 90% level of added water in the batter mixtures, exhibited the most desirable characteristics according to both instrumental and sensory analyses.

## 1. Introduction

Nowadays, there is an increasing demand for gluten-free (GF) products due to the rising prevalence of celiac disease and the growing number of individuals exhibiting symptoms of non-celiac gluten sensitivity (gluten intolerance) or wheat allergies. Gluten is the complex mixture of storage proteins in wheat flour responsible for the structure of wheat bread that gives dough distinct viscoelastic properties and baking characteristics. Replacing gluten poses a significant challenge due to its unique functionality during dough making and the baking process. Doughs made without gluten often lack cohesion and elasticity, resulting in a consistency similar to a liquid cake batter, which presents challenges during preparation and handling of the hydrated dough [1].

Unlike bread doughs containing gluten, GF bread doughs lack the three-dimensional protein–starch network, as they are typically made from pure starches or other refined flours such as maize starch and rice flour. To improve viscosity–elasticity characteristics and to ensure proper gelatinization of starch in starch-rich ingredients during baking, as well as to enhance the capacity of the dough to hold gases, significantly higher amounts of water are needed in GF formulations. This alters the dough’s consistency, making it more like a batter, and this ultimately affects the ingredient mixing, the baking process, and the overall quality of GF bread. GF batters lack elasticity and cohesion, and since there is no gluten-like protein network present, the gas retention in GF bread formulations primarily depends on the viscosity of the batter, resulting in lower specific loaf volumes [2].

The majority of GF breads, such as those made from rice flour, are often described as having an inferior mouthfeel, dry crumb, and quicker loss of freshness and lack typical flavor notes commonly present in conventional bakery items. Hence, studies in this field are focusing on quality enhancement of GF bread products [3,4]. In this context, alternative techniques and new ingredients are being employed to enhance the quality of GF bakery products [5,6,7]. Emerging baking procedures and sourdough fermentation have garnered attention due to their ability to provide textural and sensory benefits. Sourdough facilitates alterations in dough properties and enhances bread quality [7,8]. The supplementation of GF batters with sourdough results in enhanced textural attributes and increased viscosity and elasticity. These effects vary depending on the added quantity of sourdough and the specific lactic acid bacteria (LAB) employed for fermentation [9,10].

Sourdough is a mixture of flour and water fermented by native or added microflora, including LAB and yeast [11,12]. This fermentation process has been used to enhance bread quality, aiming to improve technological and quality aspects such as texture, volume, flavor, nutritional value, and shelf life of the end-product [13,14,15]. The development of the sourdough process depends on factors such as raw materials, microorganism activities, and fermentation conditions. In our previous studies, a gluten-free sourdough preparation based on an extract prepared by spontaneous submerged fermentation of chickpeas was developed [16,17]. When this sourdough was incorporated into gluten-free formulations, either as a freshly prepared or freeze-dried material, it improved bread loaf volume, textural properties, and product consumer acceptance by acting as a leavening and anti-staling agent. However, spontaneous sourdough production which relies on the native microflora of the flour can result in variable properties and quality of the fermented sourdough starter due to factors like temperature fluctuations across seasons and varying microbial composition. Therefore, the importance of targeted sourdough characteristics becomes evident to ensure consistent quality in sourdough preparations by using stable *Lactobacillus* species as starter cultures [18,19]. A common approach involves using sourdough fermented by multiple selected strains [14]. For industrial production, starter cultures containing mixed LAB and yeasts cultures are frequently employed [20,21,22].

Legume flours such as those derived from chickpeas, beans, and lentils are known for their high protein content and excellent emulsifying and foaming capacities [23,24], which enhance their water- and oil-holding capacities [25]. Moreover, phytochemicals, including phenolics and saponins, can interact with proteins, functioning as surfactants [26]. Consequently, incorporating legume-based flours into gluten-free bread formulations may improve the bread’s structure. Thus, inclusion of chickpea flour into gluten-free rice-based flour blends enhances foam properties and reduces starch retrogradation [27]. Additionally, fortification of rice-based gluten-free breads with raw or roasted chickpea flour improved loaf volume and crumb texture, retarded staling, increased protein and phenolics contents, and decreased the starch digestibility of the baked product [28,29].

The water content of bakery products plays a crucial role in dough rheology, texture, volume, taste, and flavor perception. The final quality of bakery products is affected by water mobility and distribution among the components in the composite product matrix [30]. Therefore, determining the optimal water level in the batter is essential for GF bread formulations [31]. Among GF baked goods, bread is the most widely consumed item, serving as a significant staple food globally. However, GF bread formulations typically exhibit non-desirable sensory attributes and have lower nutritional value [32,33]. This is mainly attributed to their reliance on refined flours and starches, resulting in products with high levels of available starch (rapidly digestible) that are low in micronutrients, dietary fibers, proteins, and bioactive compounds [34,35].

Therefore, the present study aimed to examine the impact of adding mixed chickpea/rice flour-based sourdough fermented by a commercial starter culture, and using various levels of water added to the batter and chickpea flour to replace part of the corn starch in the mixed rice flour/corn starch-based GF formulations, on dough and baked product quality aspects. The bread formulations were designed for consumption by individuals following a gluten-free diet, and the main objective was to improve the physicochemical and sensory properties of the GF breads. As mentioned above, most commercial GF bread products exhibit poor loaf specific volume, increased crumb hardness, and generally inferior sensory attributes. Overall, this study intended to mitigate such deficiencies and extend the shelf life of GF breads by delaying the staling process.

## 2. Materials and Methods

### 2.1. Raw Materials

Gluten-free bread formulations consisted of a flour mixture of corn starch, provided by KERAMARI BROS & CO—MANNA (Ind. area Sindos, Thessaloniki, Greece), rice flour, purchased from Piccolino (Antoniou BROS & Co., Oreokastro, Thessaloniki, Greece), and chickpea flour, prepared by grinding chickpeas (ZAKOMA SA, Thermi, Thessaloniki, Greece) twice in a hammer mill (AIRONE 121.10X, L.S.G. di Libralon Giampaolo & Gianni S.n.c., Campodarsego, Italy) and passing them through a 2 mm pore size sieve. The starter culture consisted of *Saccharomyces chevalieri*, *Lactobacillus casei*, and *Lactobacillus brevis* (LV1 sourdough starter, Lesaffre, Cerences, France). Dry baker’s yeast (*Saccharomyces cerevisiae*) was purchased from Mac Magic (ALIMENTARIA S.A., Neo Irakleio, Attiki, Greece), and a methylcellulose preparation (METHOCEL* A4C) was a product of The Dow Chemical Company (Midland, MI, USA). Other ingredients, including sunflower oil, table sugar, and salt were purchased from the local market.

### 2.2. Preparation of Sourdough and Breadmaking

For sourdough preparation, rice and chickpea flour (proportion 1:1) were mixed with an equal mass of water (flour mixture/water 1:1) at 37 °C, starter culture (2.5% flour weight basis), and sugar (2% flour weight basis) in screw-capped glass vessels and then incubated at 30 °C for 24 h in a temperature-controlled incubator (MIR-154, Sanyo Electric Co., Ltd., Ora-Gun, Gunma, Japan).

GF bread formulations made with two levels of chickpea flour, 6.5 and 10% (flour mixture basis), with or without the addition of sourdough, were used as control samples. These preparations were made with different levels of water, i.e., 80, 90, 100, and 110% (flour mixture basis), for both the sourdough and control GF breads. The flour mixture consisted of 50% rice flour and 6.5 or 10% chickpea flour, and the rest was 43.5 or 40% corn starch, respectively. All preparations also contained methylcellulose (1.5% flour mixture basis), baker’s yeast (3.0% flour mixture basis), sugar (2.5% flour mixture basis), salt (1.0% flour mixture basis), and sunflower oil (2% flour mixture basis). For the sourdough GF bread formulations, the flour mixture (rice–chickpea flours) of the sourdough constituted 14% of the final flour mixture of the batter. Overall, the composition of the various GF preparations studied is given in Table 1.

The breadmaking procedure commenced with homogenizing the solid ingredients for 5 min, followed by the addition of sunflower oil, water, and sourdough, and the batter was homogenized in a mixer (KMM023, Kenwood Major Titanium, Kenwood Ltd., Woking, UK) at low speed (160 rpm) for 10 min and at high speed (180 rpm) for an additional 5 min, followed by proofing at 37 °C and 90% RH for 30 min, and finally baking at 180 °C for 50 min. Before any analysis the GF breads were cooled for at least 2 h at room temperature and stored in sealed polypropylene bags for 2 days at 25 °C in order to evaluate staling events during storage of the baked products.

### 2.3. Evaluation of Rheological Properties of Gluten-Free Batters with Large Deformation Mechanical Measurements

The back extrusion test was conducted by a texture analyzer (Texture Analyzer-XT2i, Stable Micro Systems, Godalming, Surrey, UK) in order to evaluate the rheological parameters of the batters. The back extrusion test was conducted as described by Gidari—Gounaridou et al. [16]. Briefly, the batters were prepared as described above, and portions of 75 mL were placed in a cylindrical plexiglass (A/BE) rig with an internal diameter of 50 mm. The test involved, as a first stage, upward extrusion of the sample to 40% deformation by a 45 mm diameter flat disc plunger (A/BE45) at a speed of 1 mm/s, and, as a second stage, withdrawal of the plunger (returning the plunger to its original position). This test resulted in a force–time curve with two peaks: one positive (extrusion forces) and one negative (comprising the synergistic forces developed during plunger withdrawal). From these plots the parameters calculated using Texture Expert software (version 1.22) were hardness from the maximum force of the positive peak, consistency from the area under the curve of the positive peak, cohesiveness from the maximum force of the negative peak, and viscosity index from the area under the curve of the negative peak. The instrument was calibrated with a metal cylinder weighing 5 kg.

### 2.4. Determination of Physicοchemical Properties of Gluten-Free Batters and Breads

The pH of the sourdough (at 0 h and after 24 h of fermentation at 30 °C), batters (after mixing without baker’s yeast and after proofing with baker’s yeast), and fresh breads were determined using a pH meter (Benchtop 210, Bante Instruments, Shanghai, China). The samples were tested at a single level of added water (90%) and chickpea flour (10%).

Total titratable acidity (TTA) was expressed as the required amount of 0.1 M NaOH (mL) for pH adjustment at 8.5. A quantity of 10 g of each sample (sourdough, batter after mixing without baker’s yeast and after proofing with baker’s yeast, and bread crumb) suspended into 90 mL of double distilled water was used.

GF bread loaf volume was determined using a homemade bread volume meter, made with plexiglass [36], by the rapeseed displacement AACC 10–05.01 method [37].

A chromameter (CR-400/410, Konica Minolta, Kyoto, Japan) was used to determine color parameters according to the CIELAB system (L*, a*, b*). To calibrate the instrument, a white tile (L* = 96.9, a* = −0.04, b* = 1.84) was used. From each loaf, five measurements from the upper crust were made for each color parameter.

### 2.5. Staling Evaluation of Gluten-Free Breads

The moisture content of the bread crumb and crust was measured according to the American Association of Cereal Chemists International official methods, 44–15.02 [38].

Texture analysis of breads was performed using the Texture Profile Analysis (TPA) test with a Texture Analyzer-XT2i (Stable Micro Systems, Surrey, UK). The TPA test involves two compression cycles simulating the chewing process, using a compression flat platen probe (75 mm diameter) at a speed of 0.8 mm/s at 40% deformation and with a 5 s delay time between the two compression cycles. Each bread loaf was sliced into pieces approximately 3 cm thick, and the crumb was removed using a cylindrical bread crumb specimen mold (25 mm diameter × 30 mm height). The estimated parameters were hardness, consistency, cohesiveness, and resilience, according to Armero and Collar [39]. Measurements were conducted on the same day of breadmaking and after 1 and 2 days of storage at 25 °C for all samples, regardless of the amount of added water, except the unleavened breads with 80% added water that were excluded due to the poor loaf volume.

### 2.6. Sensory Evaluation of Gluten-Free Breads

For sensory evaluation, four samples were measured: two control samples without the addition of sourdough and two sourdough GF bread samples, each containing two levels of chickpea flour (6.5 and 10.0% flour mixture basis) at a 90% level of added water to the batter, since this was the optimum level for achieving both maximum loaf specific volume and optimum appearance of the internal structure of the crumb in the baked product. The breadmaking procedure was performed at the same day as evaluation of the baked products, using fresh samples. Twelve trained assessors evaluated the samples based on overall appearance, crumb hardness, crumb cohesiveness, overall impression, and flavor [40]. Additionally, 75 non-trained panelists assessed the samples to evaluate consumer product preference using a 9-point hedonic scale [41].

### 2.7. Statistical Analysis

Mean values for all tested parameters of at least three breadmaking trials of the GF formulations for batters and breads were analyzed by ANOVA using Tukey’s test with the IBM SPSS statistical software (version 28.0, IBM Corp., Armonk, NY, USA); pH and acidity mean values of unfermented and fermented sourdough, as well as the control and sourdough-containing breads, were compared with Dunnett’s (2-sided) test using the same software.

The parameters of the batters and breads were interrelated using the principal component analysis (PCA) by employing the IBM SPSS statistical software as well.

## 3. Results and Discussion

### 3.1. Rheological Properties of Gluten-Free Batters

GF batters cannot be subjected to the usual empirical mechanical tests of large deformations, like the typical wheat doughs, which are carried out using instruments such as farinographs, alveographs, or extensographs, due the noisy signal recorded from these instruments in testing GF batters, because of the low mechanical strength of the hydrated composite polymeric networks. A more suitable methodology to assess the rheological properties of these systems relies on tests applied to other soft materials that have high fluidity; e.g., the back extrusion test carried out by a texture analyzer, where parameters of hardness, consistency, cohesiveness, and viscosity index can be evaluated. In order to simulate possible stress–strain conditions encountered during batter preparation and batter behavior upon processing, the back extrusion test was used to evaluate the rheological properties of batters under large deformation (Table 2).

According to analysis of variance, increasing the level of added water in the batters resulted in decreased values of all the above mechanical parameters (*p* < 0.001) in a water concentration-dependent manner. However, between samples with 100 and 110% water levels, there were no significant differences. This was expected, since high levels of added water result in lower compression and resistance to flow [16]. In contrast, the addition of sourdough to the batter and the level of chickpea flour added in the mixed flour preparations did not seem to influence the rheological responses of the resultant batters (*p* > 0.05). Overall, GF batter’s rheological behavior is influenced only by the amount of added water.

### 3.2. Physicochemical Properties of Gluten-Free Batters and Breads

**Figure 1:**
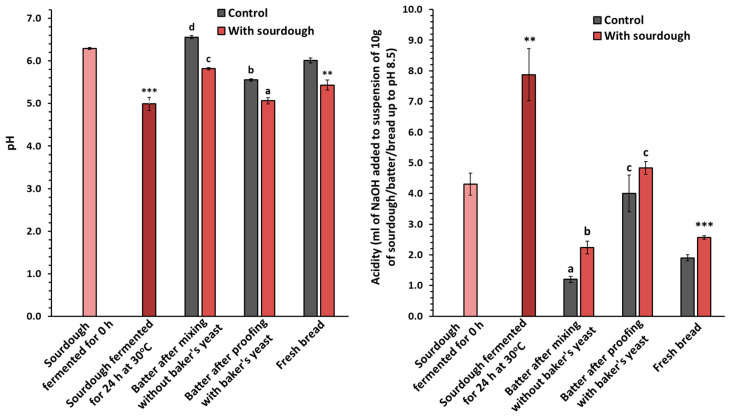
pH values and acidity of the gluten-free sourdough and control and sourdough-containing batters and breads fortified with 10% chickpea flour (flour mixture basis) and added water to the batters at a 90% level (flour mixture basis). Mean values for the batter samples followed by the same letter are not significantly different according to Tukey’s test (*p* > 0.05); mean values followed by asterisks for the sourdough fermented for 24 h and the sourdough-containing fresh bread (stored for 2 h) were significantly different (**: *p* < 0.01, ***: *p* < 0.001) when each sample was compared with the mean value of the unfermented (fermented for 0 h) sourdough and control fresh bread, respectively, using Dunnett’s (2-sided) test.

The alterations in pH values and total titratable acidity (TTA) observed after 24 h of sourdough fermentation in batters after mixing without baker’s yeast, in batters after proofing with baker’s yeast, and in fresh GF bread samples are presented in Figure 1. As expected, the pH values significantly decreased (*p* < 0.05) from 6.3 to 5.0 after 24 h of sourdough fermentation, while the TTA increased (*p* < 0.05) from 4.3 to 7.9 mL of 0.1 M NaOH. The incorporation of sourdough into the batter after mixing resulted in a pH decrease from 6.6 to 5.8, with further decreases being observed after proofing with the addition of baker’s yeast in both control and sourdough samples. This pH decrease during proofing is likely due to the activity of baker’s yeast. Between the fresh sourdough and sourdough fermented for 24 h, the latter presented the lowest pH values and the highest acidity. In general, the samples of the sourdough batters and sourdough GF bread formulations exhibited lower pH and higher TTA values than respective samples without the addition of sourdough, probably due to the production of organic acids by the starter culture used to ferment the dough mixture.

Decreased pH values and increased TTA values in the rice-based batter after proofing and in GF breads with the addition of liquid, dehydrated, and lyophilized chickpea sourdough were noted due to the production of organic acids from the spontaneous fermentation of coarsely ground chickpeas [16]. Moreover, decreased pH and increased TTA values of GF bread formulations were previously noted with the addition of chickpea/carob flour-based sourdough, where the observed differences were more pronounced with increasing sourdough levels [42].

**Figure 2:**
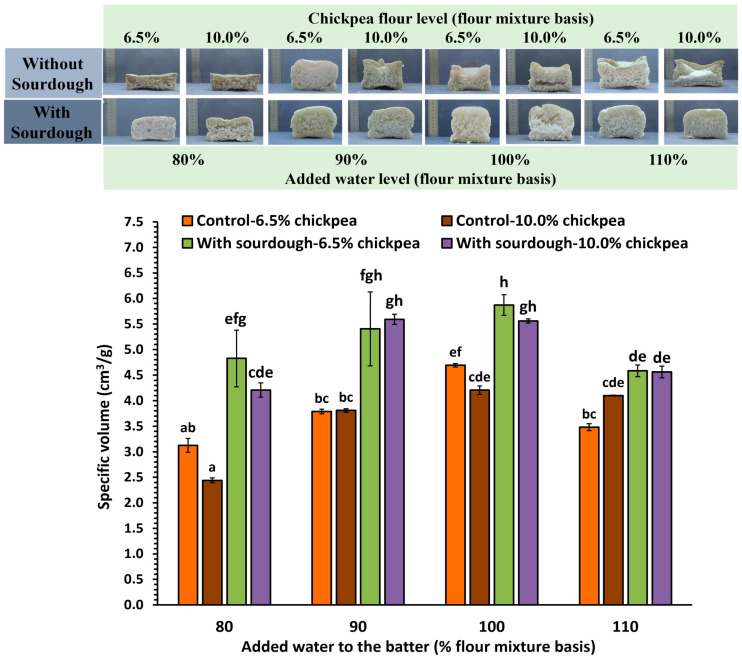
Cross-section appearance and specific volume of loaves of gluten-free control and the sourdough-containing breads, fortified with 6.5 and 10% (flour mixture basis) chickpea flour and made with different levels of added water in the batters; mean values of the different bread samples followed by the same letter are not significantly different according to Tukey’s test (*p* > 0.05).

The specific volume of bread reflects the ability of the dough to retain the gases produced during leavening, which is crucial for dough expansion and ultimately affects the acquired loaf volume, an important quality indicator for consumer preference [43]. Figure 2 illustrates the specific volumes of all treatments at various levels of added water. Regarding the effect of added water, the lowest loaf specific volumes were noted at the extreme levels of added water (80 and 110%), with optimal loaf specific volumes being noted at intermediate levels (90 and 100%). Samples with the incorporation of sourdough at both 6.5 and 10.0% chickpea flour content achieved the highest loaf specific volume at 90 and 100% levels of added water, in the range of 5.4–5.9 cm^3^/g, which is higher than the values reported for GF bread formulations studied by other researchers [44,45,46]. In GF bread formulations, water acts as a plasticizer of the composite batter/bread matrix, making the level of water added to the preparation very important. During ingredient mixing, the added water largely influences the physical properties of the expanding dough [47]. Increasing water content up to a certain level could lead to enhancement of loaf specific volume. However, with excessively high levels of added water, a lower loaf specific volume is noted due to decreasing viscosity of the batter. The impact of the combined effects of the hydrocolloids present in GF batters and water on loaf specific volume can be considered a complex phenomenon. Without the addition of a hydrocolloid with viscosity enhancement capacity in the GF formulations, incorporation of large amounts of water may also lead to excess hydration in certain areas of the composite batter, resulting in reduced stability, less gas retention, and poor loaf specific volume [44], which can explain the observations made in the present study.

Furthermore, sourdough-containing samples with a 10.0% chickpea flour concentration, at 90 and 100% added water levels, showed significant differences compared to those with 80 and 110% added water (Figure 2). In GF bread preparations without incorporation of sourdough at a 6.5% level of chickpea flour, the highest loaf specific volume was noted at a 100% added water level. Between 6.5 and 10% chickpea flour, regardless of sourdough addition to the tested samples, no significant differences were noted. Thus, the amount of chickpea flour used did not seem to influence the loaf specific volume. On the other hand, the addition of sourdough in GF bread formulations had a positive impact on this important property. The inclusion of sourdough resulted in increased values, corroborating other findings from previous studies [16,17,48,49]. Acidification induced by the sourdough activates endogenous proteases, and thus proteolysis is promoted. This process enhances interactions between water and protein, as well as water and starch molecules, thereby improving dough ductility [50].

Overall, the sourdough GF bread formulations exhibited the highest loaf specific volume regardless of the level of added water (Figure 2). Among all tested samples, the lowest loaf specific volume was noted in samples with 10% chickpea flour and an 80% level of added water (2.4 cm^3^/g), whereas the maximum was obtained for GF sourdough bread formulations with 6.5% chickpea flour at a 100% level of added water.

Analysis of variance (Table 3) indicated that variations in the chickpea flour level did not yield statistically significant effects on the specific volume of the bread loaves. Conversely, the incorporation of sourdough was found to have a positive impact. Sourdough-containing samples had an average value of 5.1 cm^3^/g compared to the control samples, which exhibited an average value of 3.7 cm^3^/g. Moreover, an increase in water content up to 100% was associated with a significant enhancement in specific volume, whereas with further addition of water, beyond 100%, there was a reduction in this quality parameter; i.e., optimal loaf specific volume was achieved at a 100% water level.

Incorporating sourdough into bread formulations resulted in increased L* values (Table 2) (*p* < 0.001). Moreover, the addition of different levels of chickpea flour significantly impacted the L* parameter (*p* < 0.001). An increase in chickpea flour concentration appeared to intensify the red hue (a* values) in the baked products, most likely due to an increased content of reducing sugars and proteins in the fortified products. This could lead to increased production of browning derivatives through the Maillard reaction [51]. The b* parameter, reflecting the yellowness of a product’s crust, also demonstrated variations concerning the influence of chickpea flour content (Table 3); samples containing 10.0% chickpea flour exhibited higher b* values (*p* < 0.001) compared to those made with 6.5% chickpea flour. Regarding the level of added water, the color parameters were also affected (<0.001), considering the role of water in both Maillard and caramelization reactions as a reactant or diluent [30].

### 3.3. Staling Kinetics of Gluten-Free Breads

Baked products generally have a limited shelf life, and the interval between production and consumption significantly impacts their quality. The shelf life of bakery products is primarily influenced by staling and microbial spoilage. Staling events, in particular, contribute to reduced consumer acceptance due to changes in crumb texture during storage, which include loss of freshness, crumb hardening, significant water loss, increase of crumbling, dropping end pieces, etc. [52,53]. Moisture is a crucial parameter in the bakery industry, as it largely impacts the texture of bakery products and, subsequently, consumer acceptance. During storage of bakery products there is moisture loss as well as redistribution of water within the product, and both are strongly linked to staling phenomena such as crumb hardening and crust softening.

In this study, the addition of sourdough, the level of water added to the batter, and the storage time were found to have a significant effect on the moisture content of the crumb (Table 4). As expected, higher levels of water added to the batter correlated with increased the crumb moisture. In contrast, the level of chickpea flour did not influence crumb moisture levels. Crumb moisture decreased after 2 days of storage at 25 °C. The incorporation of sourdough also increased crumb moisture content and decreased the rate of crumb moisture loss (Table 5), which is very important for maintaining the soft crumb texture of fortified GF breads upon storage and extending their shelf life [54]. Moreover, increased levels of water in the batter decreased the rate of moisture loss, whereas higher levels of chickpea flour in GF breads did not affect this parameter (Table 5). Similar results about the influence of the sourdough incorporation and/or the level of water added in the batter have been previously noted by other authors and were attributed to differences in the water-holding capacity of the incorporated sourdoughs [16,17,55].

Another important quality determinant is the texture of GF bread formulations. Crumb hardness was influenced by all four tested parameters; i.e., sourdough incorporation, levels of chickpea flour and added water, and storage time (Table 4). Supplemented GF breads with sourdough exhibited lower crumb hardness compared to breads without sourdough. The addition of higher levels of chickpea flour and water also contributed to a softer crumb. As expected, storage time resulted in a gradual increase in crumb hardness through the entire storage period (*p* < 0.001). It also appears that sourdough-fortified GF bread had an extended shelf life, maintaining a softer crumb texture during storage. The higher level of acidification might affect dough leavening and consequently impact crumb hardness [45]. Instead, a reduction in the level of water added to the batter and increasing storage time resulted in increased crumb hardness (*p* < 0.001). The rate of hardening was found to be significantly reduced in breads containing sourdough and those with the highest chickpea flour content (10.0%) (Table 5). This result could be attributed to the high protein content, the lower starch content, and the high fiber content of the chickpea flour, which can positively affect the staling rate in bread formulations with 10.0% chickpea flour [29,56]. Increased water levels may also result in decreased hardness and reduce the staling rate [57].

The incorporation of chickpea flour did not have a significant effect on the cohesiveness (Table 4) and the cohesiveness loss rate (Table 5) of the GF breads (*p* > 0.05). On the other hand, GF bread formulations supplemented with sourdough resulted in enhanced crumb cohesiveness (*p* = 0.001) (Table 4) as well as a reduced cohesiveness loss rate (*p* < 0.05) (Table 5). This parameter represents the internal resistance of the food’s structure [58]. Thus, high cohesiveness values are desirable to avoid typical defects of GF breads, such as crumbly crumb [29].

Crumb resilience was affected by storage time, sourdough addition, and the amount of water added, as demonstrated in Table 4. Incorporation of sourdough and an increase in water levels from 90% to 100 or 110% resulted in enhanced crumb resilience, whereas a decrease was noted with extended storage time. Resilience reflects the ability to exhibit elastic behavior [59], i.e., the capability of a material to return to its original shape after being stressed [60]. The loss of crumb resilience is a common phenomenon associated with the staling process of bakery items. Concerning the rate of resilience loss, the incorporation of sourdough, the addition of 10.0% chickpea flour, and the use of less than a 110% water level, all contributed to decreased rates of loss (Table 5).

### 3.4. Principal Component Analysis and Correlation

**Figure 3:**
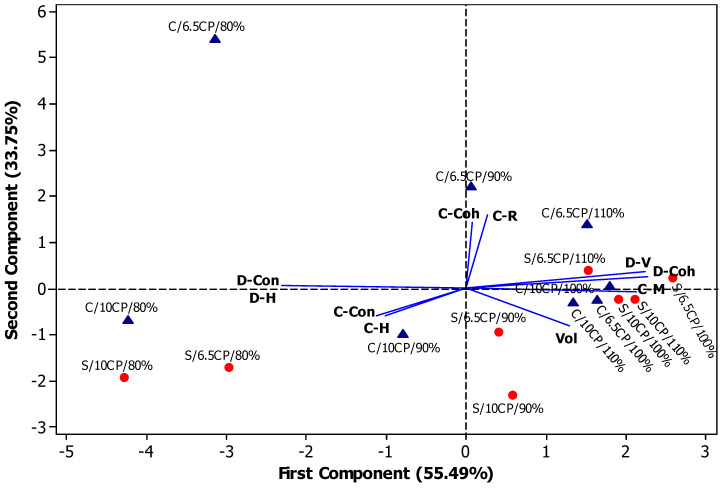
Biplot between principal component analysis and projection of the components of tested parameters. D-Con, dough consistency; D-H, dough hardness, C-Con, crumb consistency; C-H, crumb hardness; C-Coh, crumb cohesiveness; C-R, crumb resilience; D-V, dough viscosity index; D-Coh, dough cohesiveness, C-M, crumb moisture; Vol, loaf specific volume; Control (C) breads with 6.5 and 10% chickpea flour (CP) at 80, 90, 100, and 110% added water levels: C/6.5CP/80%, C/6.5CP/90%, C/6.5CP/100%, C/6.5CP/110%, C/10CP/80%, C/10CP/90%, C/10CP/100%, and C/10CP/110%; Sourdough (S) breads with 6.5 and 10% chickpea flour (CP) at 80, 90, 100, and 110% added water levels: S/6.5CP/80%, S/6.5CP/90%, S/6.5CP/100%, S/6.5CP/110%, S/10CP/80%, S/10CP/90%, S/10CP/100%, and S/10CP/110%. Red dots refer to sourdough GF bread preparations, whereas the filled blue triangles refer to control GF bread preparations.

Figure 3 presents a biplot illustrating the projection of the tested parameters in the PCA and the samples, foregrounded by the first and second principal components, which describe 55.49 and 33.75% of the variance, respectively. Consequently, the total explained variance amounts to 89.24%. Loaf specific volume, dough cohesiveness, crumb moisture, and volume are positioned at the positive end of the first component axis. Conversely, dough consistency, crumb consistency, dough hardness, and crumb hardness were located at the negative end of the first component axis, indicating a negative correlation with samples with or without the addition of sourdough, a 6.5 and 10.0% chickpea flour level, and a 100 and 110% added water level.

Crumb cohesiveness and crumb resilience were found at the positive area of the second component axis, with samples C/6.5CP/80%, C/6.5CP/90%, C/6.5CP/110%, and S/6.5CP/110% contributing most to these parameters (notation of samples as in Figure 3).

The vectors of parameters that are close to each other represent a positive and strong correlation. Thus, there is a strong positive correlation among crumb cohesiveness and crumb resilience; dough viscosity, dough cohesiveness, crumb moisture, and loaf specific volume; and crumb cohesiveness and crumb hardness. An angle of 180° between parameter vectors indicates a negative correlation. As shown in Figure 3**,** there is a negative correlation between dough viscosity, dough cohesiveness, and crumb moisture and dough consistency and dough hardness.

Quadrant I (upper right) includes the samples C/6.5CP/90%, C/6.5CP/110%, and S/6.5CP/110%, which are associated with dough viscosity and dough cohesiveness. Quadrant II (lower right) includes samples S/6.5CP90%, S/10CP/90%, C/10CP/110%, C/6.5CP/100%, S/10CP/100%, S/10CP/110%, and C/10CP/100%, which are associated with dough cohesiveness, crumb moisture, and loaf specific volume. Quadrant III (lower left) includes samples C/10CP/80%, S/10CP/80%, S/6.5CP/80%, and C/10CP/90%, associated with dough consistency, dough hardness, crumb consistency, and crumb hardness. Quadrant IV (upper left) includes the sample C/6.5CP/80%, which does not appear to be associated with any parameter.

Overall, PCA revealed that the level of added water in the batter plays a significant role in the interrelationship among the tested parameters, with the strongest connections being observed at 100 and 110% added water levels, regardless of the incorporation of sourdough and the level of chickpea flour present in the formulation.

### 3.5. Sensory Properties of Gluten-Free Breads

**Figure 4:**
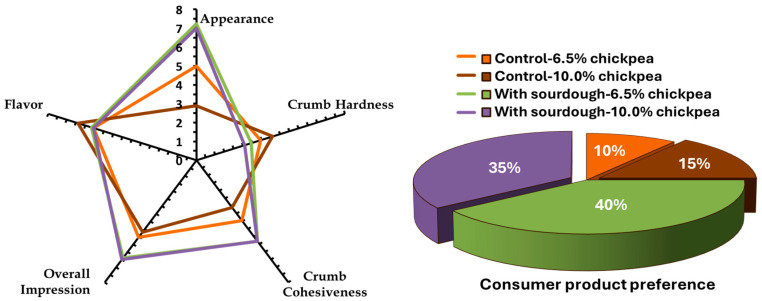
Scores of sensory attributes and consumer product preference of control and sourdough-containing breads fortified with 6.5 and 10% (flour mixture basis) chickpea flour and added water in the batters at a 90% (flour mixture basis) level; these measurements were performed on freshly baked breads (day 0 of storage).

Sensory evaluation of the bread formulations aimed to assess the sensory characteristics and record consumer preference for the tested samples. Four samples were evaluated during the sensory analysis, of which two were controls (without sourdough) and the other two were with incorporation of sourdough. For each preparation, two levels of chickpea flour (6.5 and 10.0%, flour mixture basis) at a 90% level of added water in the batter were used (Figure 4). The appearance of the breads fortified with sourdough received higher scores than those without the inclusion of sourdough. Moreover, the incorporation of sourdough in GF bread formulations resulted in a softer crumb and higher scores for crumb cohesiveness. Regarding the overall impression by the assessors, the GF sourdough bread formulations received the highest scores. Finally, the sourdough-containing samples were preferred by the majority of the consumers. Specifically, 40% of the consumers preferred the GF sourdough bread made with 6.5% chickpea flour, followed by 35% who preferred the GF sourdough bread made with 10.0% chickpea flour. Overall, it appears that the incorporation of sourdough in GF bread formulations, regardless of the chickpea flour level used, resulted in baked products with a softer and less crumbly crumb that were more attractive to consumers than the control samples. Overall, 75% of consumers preferred the GF bread formulations with the addition of sourdough.

## 4. Conclusions

In this study, a GF bread product fortified with sourdough and chickpea flour was developed. The rheological parameters of the GF batters were influenced (<0.001) only by the addition of different levels of water in the composite doughs, showing higher batter fluidity with increasing water level. Supplementation with sourdough had a positive impact on the specific volume of the bread loaves, with the highest values noted at water levels of 90 and 100%. GF bread formulations with higher chickpea flour content exhibited a more reddish crust. The batter water level and storage time had an impact on crumb moisture, with sourdough addition reducing the crumb moisture loss rate during storage. Furthermore, texture parameters and their loss rates were influenced by the incorporation of sourdough, the levels of added chickpea flour and water, and the storage time. Sourdough incorporation into batters resulted in softer, more cohesive, and more elastic bread crumb with lower staling rates. Similarly, sensory evaluation confirmed that sourdough GF formulations exhibit improved crumb texture and cohesiveness perception compared to control samples, with untrained assessors showing higher preference for the sourdough-supplemented GF breads. Overall, the results suggest that the addition of a rice/chickpea flour-based sourdough fermented by a commercial starter culture and the use of optimal water levels in GF batters can improve the quality characteristics and shelf life of GF breads, making them more appealing to consumers.

## Figures and Tables

**Table 1:** Formulations of control and sourdough-supplemented gluten-free breads made at two different levels of chickpea flour and four different levels of added water into the batter formulation.

	Control Breads								Sourdough Breads							
Level of Chickpea Flour (Total) ^1^	6.5				10.0				6.5				10.0			
Level of Added Water (Total) ^2^	80.0	90.0	100.0	110.0	80.0	90.0	100.0	110.0	80.0	90.0	100.0	110.0	80.0	90.0	100.0	110.0
Added ingredient ^1^																
Rice flour	50.0	50.0	50.0	50.0	50.0	50.0	50.0	50.0	50.0	50.0	50.0	50.0	50.0	50.0	50.0	50.0
Corn starch	43.5	43.5	43.5	43.5	40.0	40.0	40.0	40.0	43.5	43.5	43.5	43.5	40.0	40.0	40.0	40.0
Chickpea flour ^3^	6.5	6.5	6.5	6.5	10.0	10.0	10.0	10.0	-	-	-	-	3.5	3.5	3.5	3.5
Sourdough (rice flour, chickpea flour, water) ^3^	-	-	-	-	-	-	-	-	26.0	26.0	26.0	26.0	26.0	26.0	26.0	26.0
Water ^3^	80.0	90.0	100.0	110.0	80.0	90.0	100.0	110.0	67.0	77.0	87.0	97.0	67.0	77.0	87.0	97.0
Methylcellulose	1.5	1.5	1.5	1.5	1.5	1.5	1.5	1.5	1.5	1.5	1.5	1.5	1.5	1.5	1.5	1.5
Baker’s yeast	3.0	3.0	3.0	3.0	3.0	3.0	3.0	3.0	3.0	3.0	3.0	3.0	3.0	3.0	3.0	3.0
Sugar	2.5	2.5	2.5	2.5	2.5	2.5	2.5	2.5	2.5	2.5	2.5	2.5	2.5	2.5	2.5	2.5
Salt	1.0	1.0	1.0	1.0	1.0	1.0	1.0	1.0	1.0	1.0	1.0	1.0	1.0	1.0	1.0	1.0
Sunflower oil	2.0	2.0	2.0	2.0	2.0	2.0	2.0	2.0	2.0	2.0	2.0	2.0	2.0	2.0	2.0	2.0

^1^ Expressed as % of total weight of flour mixture (rice flour + corn starch + chickpea flour); i.e., total weight basis of rice flour, corn starch, and chickpea flour mixture included into both the batter and the sourdough preparation. ^2^ Expressed as % of total weight of flour mixture present in the batter and the sourdough. ^3^ Expressed as % of flour mixture weight basis and incorporated into the batter.

**Table 2:** Analysis of variance and significance (*p*-values) of the general linear model main effects for the rheological parameters derived from back extrusion testing of gluten-free control and sourdough-containing batters, fortified with chickpea flour and made with different levels of added water.

			Rheological Parameters of Batters		
Factors	Levels	Hardness (Ν)	Consistency (Ν·mm)	Cohesiveness (Ν)	Viscosity Index (Ν·mm)
Sourdough	Control	5.64 a ^3^	73.94 a	3.87 a	26.60 a
	With sourdough	4.94 a	65.03 a	4.49 a	32.30 a
	*p*-value ^2^	0.631	0.633	0.610	0.470
Chickpea flour (% f.m.b.) ^1^	6.5	4.71 a	63.03 a	3.76 a	25.70 a
	10.0	5.87 a	75.94 a	4.59 a	32.66 a
	*p*-value ^2^	0.431	0.490	0.500	0.421
Added water (% f.m.b.) ^1^	80	13.16 c	169.54 c	10.78 c	74.78 c
	90	4.17 b	64.01 b	3.81 b	26.83 b
	100	2.09 a	28.43 a	1.35 a	9.51 a
	110	1.19 a	15.96 a	0.76 a	5.58 a
	*p*-value ^2^	<0.001	<0.001	<0.001	<0.001

^1^ f.m.b.: flour mixture basis. ^2^ The main effects of the tested factors are significant for *p* < 0.05. ^3^ Mean values for the specified rheological parameter at a certain level of one factor were calculated using all levels of the other factors. Values followed by the same letter in the same column and for the same factor are not significantly different (*p* > 0.05); comparison of means was made by Tukey’s test.

**Table 3:** Analysis of variance and significance (*p*-values) of the general linear model main effects for the loaf specific volume and crust color parameters of gluten-free control and sourdough-containing breads fortified with chickpea flour and made with different levels of added water in the respective batters.

Factors	Levels	Specific Volume (cm^3^/g)	Crust Color Parameters ^4^		
			L*	a*	b*
Sourdough	Control	3.69 a ^3^	60.15 a	3.72 b	28.93 a
	With sourdough	5.07 b	69.39 b	1.01 a	29.12 a
	*p*-value ^2^	<0.001	<0.001	<0.001	<0.001
Chickpea flour (% f.m.b.) ^1^	6.5	4.46 a	66.26 b	1.01 a	27.53 a
	10.0	4.31 a	63.28 a	3.72 b	30.49 b
	*p*-value ^2^	0.053	0.053	<0.001	<0.001
Added water (% f.m.b.) ^1^	80	3.65 a	66.05 c	3.07 c	30.58 c
	90	4.62 c	63.81 ab	2.54 bc	28.69 b
	100	5.08 d	65.95 bc	2.20 ab	29.25 b
	110	4.18 b	63.26 a	1.64 a	27.52 a
	*p*-value ^2^	<0.001	<0.001	<0.001	<0.001

^1^ f.m.b.: flour mixture basis. ^2^ The main effects of the tested factors are significant at *p* < 0.05. ^3^ Mean values for each specified color parameter or specific volume at a certain level of one factor were calculated using all levels of the other factors. Values followed by the same letter in the same column and for the same factor are not significantly different (*p* > 0.05); comparison of means was made by Tukey’s test. ^4^ L*, a* and b*: Color parameters of CIE system.

**Table 4:** Analysis of variance and significance (*p*-values) of the general linear model main effects for the moisture and textural parameters of the crumb of gluten-free control and sourdough-containing breads fortified with chickpea flour and made with different levels of added water in the respective batters; breads were stored at 25 °C up to 2 days.

Factors	Levels	Crumb Moisture Content (% *w*/*w*)	Crumb Textural Parameters ^4^		
			Hardness (N)	Cohesiveness	Resilience
Sourdough	Control	46.26 a ^3^	0.98 b	0.50 a	0.24 a
	With sourdough	49.57 b	0.83 a	0.53 b	0.25 b
	*p*-value ^2^	0.005	0.005	0.001	0.003
Chickpea flour (% f.m.b.) ^1^	6.5	48.20 a	1.01 b	0.51 a	0.25 a
	10.0	47.64 a	0.79 a	0.51 a	0.24 a
	*p*-value ^2^	0.460	0.459	0.820	0.578
Added water (% f.m.b.) ^1^	80	41.88 a	1.34 c	0.51 ab	0.23 a
	90	46.39 b	0.90 b	0.42 a	0.22 a
	100	56.72 c	0.64 a	0.53 b	0.25 b
	110	52.68 d	0.93 b	0.52 ab	0.26 b
	*p*-value ^2^	<0.001	<0.001	0.001	<0.001
Storage time (d)	Day 0	50.19 c	0.50 a	0.76 c	0.38 c
	Day 1	48.44 b	0.74 b	0.43 b	0.19 b
	Day 2	45.13 a	1.45 c	0.35 a	0.15 a
	*p*-value ^2^	<0.001	<0.001	<0.001	<0.001

^1^ f.m.b.: flour mixture basis. ^2^ The main effects of the tested factors are significant at *p* < 0.05. ^3^ Mean values of each specified parameter at a certain level of one factor were calculated using all levels of the other factors. Values followed by the same letter in the same column and for the same factor are not significantly different (*p* > 0.05); comparison of means was made by Tukey’s test. ^4^ Estimated by Texture Profile Analysis (TPA).

**Table 5:** Analysis of variance and significance (*p*-values) of the general linear model main effects for the change rate of moisture and textural parameters of crumb of gluten-free control and sourdough-containing breads fortified with chickpea flour and made with different levels of added water in the respective batters; breads were stored at 25 °C up to 2 days.

Factors	Levels	Change Rate ^4^			
		Crumb Moisture Content (g H_2_O/100 g Crumb/d)	Crumb Textural Parameter ^5^		
			Hardness (N/d)	Cohesiveness (1/d)	Resilience (1/d)
**Sourdough**	Control	3.96 b ^3^	0.61 b	0.25 b	0.14 b
	With sourdough	1.11 a	0.35 a	0.19 a	0.11 a
	*p*-value ^2^	<0.001	<0.001	<0.050	<0.001
**Chickpea flour** **(% f.m.b.) ^1^**	6.5	2.34 a	0.59 b	0.21 a	0.13 b
	10.0	2.72 a	0.34 a	0.22 a	0.11 a
	*p*-value ^2^	0.327	<0.005	0.665	<0.001
**Added water** **(% f.m.b.) ^1^**	80	4.50 c	0.70 b	0.20 a	0.11 a
	90	2.50 b	0.50 ab	0.20 a	0.11 a
	100	2.26 ab	0.26 a	0.23 a	0.12 a
	110	0.87 a	0.51 ab	0.20 a	0.13 b
	*p*-value ^2^	<0.001	<0.050	0.835	<0.050

^1^ f.m.b.: flour mixture basis. ^2^ The main effects of the tested factors are significant at *p* < 0.05. ^3^ Mean values of each specified parameter at a certain level of one factor were calculated using all levels of the other factors. Values followed by the same letter in the same column and for the same factor are not significantly different (*p* > 0.05); comparison of means was made by Tukey’s test. ^4^ Calculated from the slope of the linear regression model fitted to the data of the crumb moisture content or the textural parameter versus storage time. ^5^ Estimated by Texture Profile Analysis (TPA) testing.

## Data Availability

The original contributions presented in the study are included in the article, further inquiries can be directed to the corresponding author.
